# Clinicians’ perceptions of a potential wearable device for capturing upper limb activity post-stroke: a qualitative focus group study

**DOI:** 10.1186/s12984-021-00927-y

**Published:** 2021-09-08

**Authors:** Lisa A. Simpson, Carlo Menon, Antony J. Hodgson, W. Ben Mortenson, Janice J. Eng

**Affiliations:** 1 Graduate Program in Rehabilitation Sciences, University of British Columbia, Vancouver, Canada; 2 Biomedical and Mobile Health Technology Laboratory, Department of Health Sciences and Technology, ETH Zurich, Zurich, Switzerland; 3 Department of Mechanical Engineering, University of British Columbia, Vancouver, Canada; 4 Department of Occupational Sciences and Occupational Therapy, University of British Columbia, Vancouver, Canada; 5 Department of Physical Therapy, University of British Columbia, 212-2177 Wesbrook Mall, Vancouver, BC V6T 1Z3 Canada

**Keywords:** Stroke, Wearable technology, Upper limb, Qualitative

## Abstract

**Background:**

There is growing interest in the use of wearable devices that track upper limb activity after stroke to help determine and motivate the optimal dose of upper limb practice. The purpose of this study was to explore clinicians’ perceptions of a prospective wearable device that captures upper limb activity to assist in the design of devices for use in rehabilitation practice.

**Methods:**

Four focus groups with 18 clinicians (occupational and physical therapists with stroke practice experience from a hospital or private practice setting) were conducted. Data were analyzed thematically.

**Results:**

Our analysis revealed three themes: (1) “Quantity and quality is ideal” emphasized how an ideal device would capture both quantity and quality of movement; (2) “Most useful outside therapy sessions” described how therapists foresaw using the device outside of therapy sessions to monitor homework adherence, provide self-monitoring of use, motivate greater use and provide biofeedback on movement quality; (3) “User-friendly please” advocated for the creation of a device that was easy to use and customizable, which reflected the client-centered nature of their treatment.

**Conclusions:**

Findings from this study suggest that clinicians support the development of wearable devices that capture upper limb activity outside of therapy for individuals with some reach to grasp ability. Devices that are easy to use and capture both quality and quantity may result in greater uptake in the clinical setting. Future studies examining acceptability of wearable devices for tracking upper limb activity from the perspective of individuals with stroke are needed.

**Supplementary Information:**

The online version contains supplementary material available at 10.1186/s12984-021-00927-y.

## Background

The majority of individuals who have a stroke experience upper limb impairment [1] and many of these individuals will have persistent difficulties using their limb in daily activities [2]. Animal studies suggest thousands of challenging repetitions are necessary to drive functional recovery [3]. Although the optimal dose of upper limb repetitions in humans is largely unknown, studies suggest that greater amount of movement repetitions of the upper limb are associated with greater functional improvements [4]. This is in contrast to the typical doses of movement practice during stroke rehabilitation which are low and do not come close to those obtained in animal studies that demonstrate significant recovery [5]. In addition, functional improvements made during rehabilitation do not always translate into greater use of the limb in daily life [6]. This is also of concern as decreased use in daily life could jeopardize the functional gains made during costly rehabilitation hospital stays and hamper any future functional recovery.

Wearable technology that monitors and provides feedback on how much the upper limb is moving may help determine a more specific relationship between number of repetitions and recovery and may also motivate individuals to achieve higher levels of movement repetitions. A growing interest in wearable systems that monitor upper limb movement post-stroke has already resulted in multiple review papers describing these technologies and summarizing their research [7–9]. The authors of these review papers agree there is a potential for technologies to make a positive contribution in the field of upper limb rehabilitation post-stroke however they also recognize challenges exist to their clinical use. Although the authors provide some nice suggestions to increase the clinical utility and uptake of these technologies, it is also important to understand the needs and views of clinicians to either inform the development of new technologies or to increase adoption of existing technology. Indeed, a user-centered design approach is optimal for obtaining relevant information that could inform the design process and/or potentially impact future knowledge translation activities [10]. Three qualitative or survey-based studies have previously examined healthcare providers’ perceptions of specific upper limb technologies used following stroke such as robotic wearable devices, functional electrical stimulation and virtual reality [11–13]. Although these studies revealed important considerations for the design of upper limb technology in stroke rehabilitation in general, none of these studies specifically examined perceptions of wearable devices for the purpose of tracking upper limb activity. Thus, the purpose of this study was to understand clinician perceptions of a potential wearable device for capturing upper limb activity following stroke and its use within rehabilitation practice.

## Methods

This study used a qualitative description approach to obtain comprehensive descriptions of clinicians’ experiences working with the upper limb and perceptions of a potential wearable device for capturing upper limb activity post stroke [14]. Focus groups were used as the primary method of data collection as they are able to obtain views from groups of people with both shared (i.e., work in same institution) and varied (i.e., work with clients at different stages of recovery) experiences in a time efficient manner [15]. All participants provided written informed consent prior to the start of the focus group sessions. Ethical approval for this study was provided by the local university ethics board and the Standards for Reporting Qualitative Research (SRQR) was used to report the findings.

### Participants

Participants were a convenience sample of physical and occupational therapists recruited from a local rehabilitation hospital and a neurorehabilitation physical therapy private practice clinic who responded to a recruitment email and who met the inclusion criteria. Clinicians recruited from the rehabilitation hospital worked in inpatient, outpatient and/or community rehabilitation. Participants were eligible if they were 19 years old or greater and had at least one year experience working with individuals with stroke. All individuals who expressed interest in participating and met the inclusion criteria were included.

### Procedures

Four focus groups were conducted with three to five participants each (18 clinicians in total) from March 2015 to April 2015. Sessions were conducted at the clinicians’ workplaces and lasted between 45 to 90 min. Three sessions were conducted at the local rehabilitation hospital and included both occupational therapists and physical therapists. One session conducted at the private neurorehabilitation physical therapy clinic only included physical therapists. A value stipend was offered to all participants. The same moderator (LAS) facilitated discussion for all group sessions using a semi-structured interview guide. In addition, a research assistant or an engineer working on the wearable sensor research team acted as an observer at each session. The observer took notes and assisted in summarizing and/or clarifying participants’ statements. The semi-structured focus group guide was developed and reviewed by the research team, which included individuals from the following fields: engineering, physical therapy, occupational therapy, and stroke research. The interview guide comprised open-ended questions and more specific probing questions to generate discussion around the following topics: (1) participants’ experiences working with the upper limb; (2) initial thoughts about using a wearable device to capture upper limb movement in their practice; (3) important movements for an ideal device to capture or not capture and (4) opinions about a specific prototype that captured the number of grasp and release movements. The interview guide can be found in Additional file 1: Appendix S1. The moderator and observer debriefed immediately after each session and discussed what they heard and their reactions. All focus group sessions were audio-recorded, transcribed verbatim and reviewed for accuracy. Summaries of the group discussions were emailed to all focus group participants for their review. A total of 8 participants responded to the email request. One participant suggested the summary should include a further point of discussion while the other seven confirmed the summary accuracy without further comments.

### Research team positionality and reflexivity

The primary author (LAS) is a female occupational therapist and PhD student with prior experience conducting qualitative focus groups and treating the upper limb after stroke. CM is a male professor in health sciences and technology and has extensive research experience in the design of biomedical technologies. AJH is a male professor in mechanical engineering with extensive research experience in the field of biomechanics, neuromotor control and the development of biomedical technologies. WBM is a male associate professor in occupational therapy and has extensive qualitative research experience in the area of assistive technology. JJE is a female professor in physical therapy with extensive experience in the design of clinical interventions post stroke. LAS, CM, AJH and JJE were part of a wearable device research team planning development of a wearable device using technology that could capture activity of the hand. This intention might have led to a greater amount of time discussing participants’ opinions about a potential device that captured grasp and release function. However, questions regarding participants’ current experiences treating the upper limb and their opinions about an ideal device for capturing upper limb activity were sought before a specific prototype was introduced.

### Data analysis

Transcripts were analyzed thematically [16]. The focus group transcriptions were first read in full by the two individuals coding the data and then transferred into Excel spreadsheets for coding. One of the authors (LAS) and a research assistant who had experience conducting qualitative research coded the first two focus groups independently by labelling segments of the data (ie. information contained within an Excel cell). They then worked collaboratively to generate an initial coding guide. The initial coding guide was then applied to the remaining two focus groups by LAS who refined the coding guide and applied it to all four focus group sessions. The final coding guide can be found in Additional file 2: Appendix S2. LAS and JJE then generated initial themes and subthemes independently by doing the following: (1) looking for patterns of meaning across the codes to form subthemes; (2) looking for patterns of meaning across subthemes to form themes and (3) checking the potential themes against the dataset to see if they accurately described the data. These two authors then worked collaboratively to generate the final themes and subthemes. Participants were given a participant number code (in the form of P#) to anonymize any excerpts provided in the manuscript. Patterns in the practice context (ie. occupational/physical therapist, public/private setting, inpatient/outpatient therapist) were examined when differences in experience/opinions were noted. It should be noted that our analysis approach bears similarities to, but is not entirely congruent with, reflexive thematic analysis described by Braun and Clarke. [16] In that regard, although we did use a coding guide, we kept it fluid throughout the research process.

The following strategies were used to promote trustworthiness: participant checks, triangulation and reflexivity. As noted above, participant checks were conducted by providing summaries of focus group discussions to all participants. Researcher triangulation consisted of using a multidisciplinary research team and participant triangulation occurred via recruitment of clinicians in different settings (public and private) and who work with clients at different stages of their recovery (inpatient rehabilitation to chronic). Reflexivity was facilitated through debriefing sessions at the end of each session.

## Results

Table 1 outlines participant characteristics of the 18 clinicians who participated in the focus group sessions. Analysis of the four focus group sessions identified three themes and 7 subthemes (Table 2).

**Table 1:** Participant characteristics

Characteristic	N = 18
Sex: Female, N (%)	16 (88.8%)
Years working with individuals with stroke, mean (SD)	7 (5.6)
Profession, N(%)	
Physical therapist	11 (61.1%)
Occupational therapist	7 (38.9%)
Primary setting, N(%)	
Inpatient rehabilitation	10 (55.6%)
Outpatient rehabilitation	7 (38.9%)
Both	1 (5.5%)

**Table 2:** Main themes and subthemes

Theme 1: Quantity and quality is ideal Subtheme1: Movement quantity Subtheme 2: Movement quality Subtheme 3: Identifying specific movements
Theme 2: Most useful outside therapy sessions Subtheme 1: Monitor quantity and quality Subtheme 2: Training and promote carry over
Theme 3: User-friendly please Subtheme 1: Client specific Subtheme 2: Easy to use

### Theme 1: quantity and quality is ideal

All therapists wanted a sensor that would capture both movement quantity and quality of movement. In subtheme 1 (Movement Quantity) however, most therapists also stated that a device that only captured movement quantity would still be beneficial for a specific set of clients. For instance, one therapist stated (P15): “…..*at this point I don’t even use any devices, …if I had a device that just tracked how much this person is using that arm, then that’s a start.*”

Therapists described clients who might use a device that only captured quantity as *“high functioning (P15),”* “*having good control and just not using it (P8),*” possessing “*functional use (P2)*” or having “*decent muscle activation through the whole limb (P5).*” As P1 indicated, this device would be beneficial for individuals who *“have pretty good movement and maybe not terrible compensatory movements … but they’re at risk for learned non-use.*”

The importance of movement quality was salient across all the focus groups and thus formed subtheme 2 (Movement Quality). Therapists described how their practice focused on promoting more isolated and normal movement patterns and reducing compensations for individuals who had some use of their affected upper limb. They expressed a need to avoid some movement patterns or spasticity when prescribing homework tasks. For instance, P7 stressed the importance of movement quality when describing an example of movement instructions provided to clients:

“I want you to move your arm, but while holding an object with your wrist back and arm extended right? … so you’re gonna practice doing this exercise, but in this specific way.”

Therapists described how a device that could capture movement quality aligned with how they practice. For instance, P16 stated:

“in stroke, there are certain patterns that people move in, and part of what we want to do is to break those patterns and try to create more specific movements. So to be able to see, […] the change in, […] the coordination and what not, it would be really good.”

Referring to the ability of a device to distinguish between desirable (normal) and undesirable (compensatory) movement patterns, P10 stated: “*it could be a very powerful tool if it had the ability … to distinguish the two and give people feedback on a … more normal movement pattern.* Some therapists indicated that an ideal wearable device would provide some biomechanical information such as muscle activation and joint movement patterns. One therapist stated (P13):

“Well I’m just thinking, [the device] can hopefully detect angles and all that kind of stuff. Could it actually put all those angles and the speed and everything together, to recreate ……and you can get their overall pattern in general, of how they reach and grasp.”

Therapists in all group sessions also expressed a desire to distinguish between voluntary and involuntary movement, as well as between functional and non-functional movements when discussing an ideal wearable device for capturing upper limb activity. As P13 questioned:

“…is it truly volitional, where they’re trying to initiate it, or is it more of a spasm? […], While they’re eating, are they truly trying to bring their cup to their mouth, or do they suddenly get spasms in some way, or they’re just going into more of a flexor synergy?”

Moreover, maximizing functional use of the upper limb was a goal frequently identified by therapists across all focus groups. As P13 summarized, “*I think the number one focus is always incorporate* [the affected arm] *into function.*” Finally, two therapists who saw their clients daily in an inpatient setting questioned whether a device that did not capture quality in some manner would be useful. As one P5 stated, “*it can’t just be the number, right, so it has to be some sort of a way to qualify* [the movement].”

In subtheme 3 (Identifying Specific Movements), therapists had a difficult time identifying a core set of movements they felt a wearable device should capture. Some of the focus groups spent more time debating particular movements of interest than others; however ultimately most therapists agreed that it depended on the client. As P9 stated:

“….it really depends on the person, […] you may have someone who you really want to focus on the hand, but then for other people, like a lot of people, […] I’m focusing, because they don’t have any movement, and focusing more elbow, shoulder”

Despite expressing difficulty in prioritizing one movement/joint over another, common movements of interest were identified across the groups. Table 3 provides a list of the movements identified as important with associated group session excerpts. The movements/joints of interest are organized into the following three major areas: shoulder, reach to grasp and extension).

**Table 3:** Upper limb movements/joints of interest identified by participants

Movement/Joint of Interest	Focus group session excerpts
Shoulder complex	*“So my primary goal, probably for all level[s] though is scapular stabilization… even if they do have a better presenting arm, usually it’s still coming back to scapular stabilization and setting.*”-P8
Reach to grasp	*“And is it with you know, a nice neutral wrist or 30 degrees of wrist extension or are they releasing things by letting the wrist flex […]. You know when they’re reaching, is it a combination of […] anterior delt, shoulder flexion and elbow extension through their triceps, with, you know, the shoulder in fairly neutral alignment”* -P13
Extension (at elbow, wrist and fingers):	“*we see this all the time you know we see into flexion, into internal rotation, we see that more often than we see … extension, period*.”-P3 “….*something that could read […], so you know that they’re doing something out here* [showing elbow extension] *rather than here”*-P17 *“This is a bit off-topic… but there are a lot of people who rest in certain postures for […] the majority of the day. And if it did have something that reminded you […] “your wrist has been flexed for 3 h*”-P18 *“To be honest, […] most of the struggle with neurological clients is not the grasp, because that’s a flexor pattern that comes back in someone who is getting any amount of motor control. This is the hard part* [showing extension of the fingers] *… that’s what we spend most of our time working on…”*-P2

### Theme 2: most useful outside therapy sessions

Therapists indicated that a wearable device would only be useful when worn outside of therapy. One therapist described why she wouldn’t use the device during therapy sessions (P18):

“I’m not going to get the number of reps they need in my hour session to make a huge change. This is me teaching the movement. So I’m more worried- like we get the quality down and you do the quantity at home.”

In subtheme 1 (Monitor Quantity and Quality), therapists described how they would use a wearable device to monitor quality and quantity outside of therapy. Therapists who worked with individuals who lived in the community discussed the potential to use a wearable device as a way to monitor adherence to an exercise program. Some benefits to monitoring clients’ movement quantity outside of therapy identified by therapists were that it would help with clients who may not be “*accurate historians*” and it could also assess whether amount of practice was a factor in clients’ progression. For instance, P16 stated: *“So if they’re getting the practice done, but they’re not changing, then we need to do something else.”* Not all therapists agreed that a wearable device should be used to monitor adherence to an exercise program, however. Therapists in one focus group who consisted mainly of inpatient therapists stated they wouldn’t use a device to monitor clients’ adherence to homework. As one inpatient therapist stated (P3), *“Yeah, because if you give people 2 reps of 10 than they generally will just do it.”* Therapists suggested, however, that a beneficial use for a wearable device to track upper limb activity would be to provide clients with concrete information about their use to promote self-monitoring or to act as an outcome measure to capture change in activity levels over the course of therapy. Therapists shared that individuals with stroke are not always aware of how much they use their arm. As P1 explained:

“Well and it is also something that’s not as easy to track so if … say at the beginning of their hospitalization or rehab stay, it was worn for X amount of days, and you said ok this day we got twenty whatever, and then you measure it the following week and you say ok you’ve got thirty this week so you can actually show an improvement or… maybe not. It’s something that’s an objective measure that is helpful for both therapist and clients. ”

In discussions about what their practice looked like before their opinions about a wearable sensor were sought, many therapists described the challenges with prescribing homework and promoting upper limb practice outside of their therapy sessions. They discussed the inherent frustration with doing task practice with the affected limb, the potential to overwhelm the patient from prescribing too many exercises and the need to provide very specific guidelines for some clients. P3 discussed the variation in exercise prescription in the following comment:

“I find some other clients it’s more.. it needs to be a bit more structured. ..so thirty minutes a day… you’re going to pick four to five out of a list of tasks, and to give them a bit more of intensity, that intense practice, [whereas] making that conscious effort to, incorporate it,[…] some people can do that. ”

For some inpatient therapists, homework was sometimes seen as a futile task. As P6 explained: “*I don’t find a lot of my clients do their homework, let’s be honest […]. I don’t find giving homework very effective”.*

In subtheme 2 (Training and Promote Carry Over), therapists discussed using the device for training purposes when outside of therapy sessions. For instance, therapists expressed enthusiasm about using the device as a biofeedback tool. Biofeedback was described by P18 as: *“…if it had something that said […]ok we’re keeping this wrist neutral for […] 5 out of those 10 contractions. Then that would be useful to us*.” Biofeedback was envisioned by P6 as: *“if it […] beeped every time you hit the right spot […]. Then at least they have an external feedback to […] what I’m looking for.”* Providing information to individuals with stroke about their quality of use was described as a way the devices could promote carry over from clinic to home, as described by P10: “*To augment, […] what we’re doing in therapy,[…] like use it as a tool to sort of extend that”.*

Finally, therapists discussed the potential of a wearable device to motivate greater use of the affected limb. As P17 explained:

“So I think it can set […] little small goals that are achievable for them each day. Because sometimes just using their arm more, or sometimes the task- they might want to use their arms so far down the road that having it broken down might be motivating for them.”

Thus, therapists envisioned using the information captured by a device to set movement goals for clients to work towards to increase their movement practice.

### Theme 3: user-friendly, please

The final theme centered around features that might increase the usability of a future upper limb wearable device. In the first subtheme, therapists highlighted the importance of client specificity in their practice and in the design of a wearable device. Therapists described how factors such as motor impairment, cognition and levels of fatigue affected what goals they created, how homework was assigned and how functional use of the affected upper limb was promoted. When discussing some of the UL goals they create, P12 noted: “*I think that, yeah it does definitely obviously vary with the client and how much motor recovery they have at the moment……”* Level of cognition and fatigue were primarily mentioned by inpatient therapists when discussing what homework tasks to provide. As P13 stated:

“Especially early on with some of the inpatient clients that cognitive level is a huge factor in terms of how much you would decide to provide to that client, because if they don’t have cognitive ability to follow through well, then we’re not going to do that; unless they have maybe a really reliable family member who can guide the person.”

One therapist referred to cognitive level of clients when considering who should be given a wearable device at all (P2):

“And then you would have to only limit it to […] clients who are cognitively capable. Who need it physically but cognitively capable of making goals and motivated to achieve them. The pool just gets smaller.”

Other identified client related factors that impact participants’ practice were time post stroke, how often clients were being seen, family dynamics and client goals. Inpatient therapists highlighted that individuals were seen daily by therapists when discussing homework whereas outpatient therapists indicated that they had less time with a client. One therapist described how this fact may be related to the appeal of using a wearable device to monitor use in the following statement (P11):

“…so not only is it an outcome measure tool for the therapist, but for the client to be able to self-monitor and see their own progress […], especially for outpatients I think right, because we only see people once or twice a week so we have no clue what they’re doing the other five days. At least on inpatients […] you guys see them five days a week.”

In subtheme 2, therapists indicated that technology used in their practice should be easy to use for both the therapists and clients. When imagining the features that an ideal wearable device for the upper limb might possess, many therapists expressed a preference for one which could be easily calibrated or programmed to meet the needs of any given individual.

“You could program, when you get it to this person, [..] four movements that it is looking for only. […] instead of creating ten different devices that measure ten different things, it’s […] a device that measures all those, but you can program which ones you’re looking for. (P15)”

In one focus group, therapists expressed their frustration with devices that have multiple steps involved in the set-up and highlighted the importance of simple set up procedures. As P7 explained: “*I think it has to be easy […] from the very first time [….] not when you’re an expert.*” Another therapist stated the device should not interfere with a client’s function in the following statement (P11): “*It’s either going to help the person be actually more aware and try to use it more, or could actually hinder the person depending on what the actual device looks like…”.*

Finally, P8 identified the significance of ease of use in the following comment: “.… *things have to be easy to apply and easy to turn on, because they just don’t get used in therapy*” Thus, therapists highlighted the potential impact ease of use plays in the clinical uptake of any device.

## Discussion

**Fig. 1:**
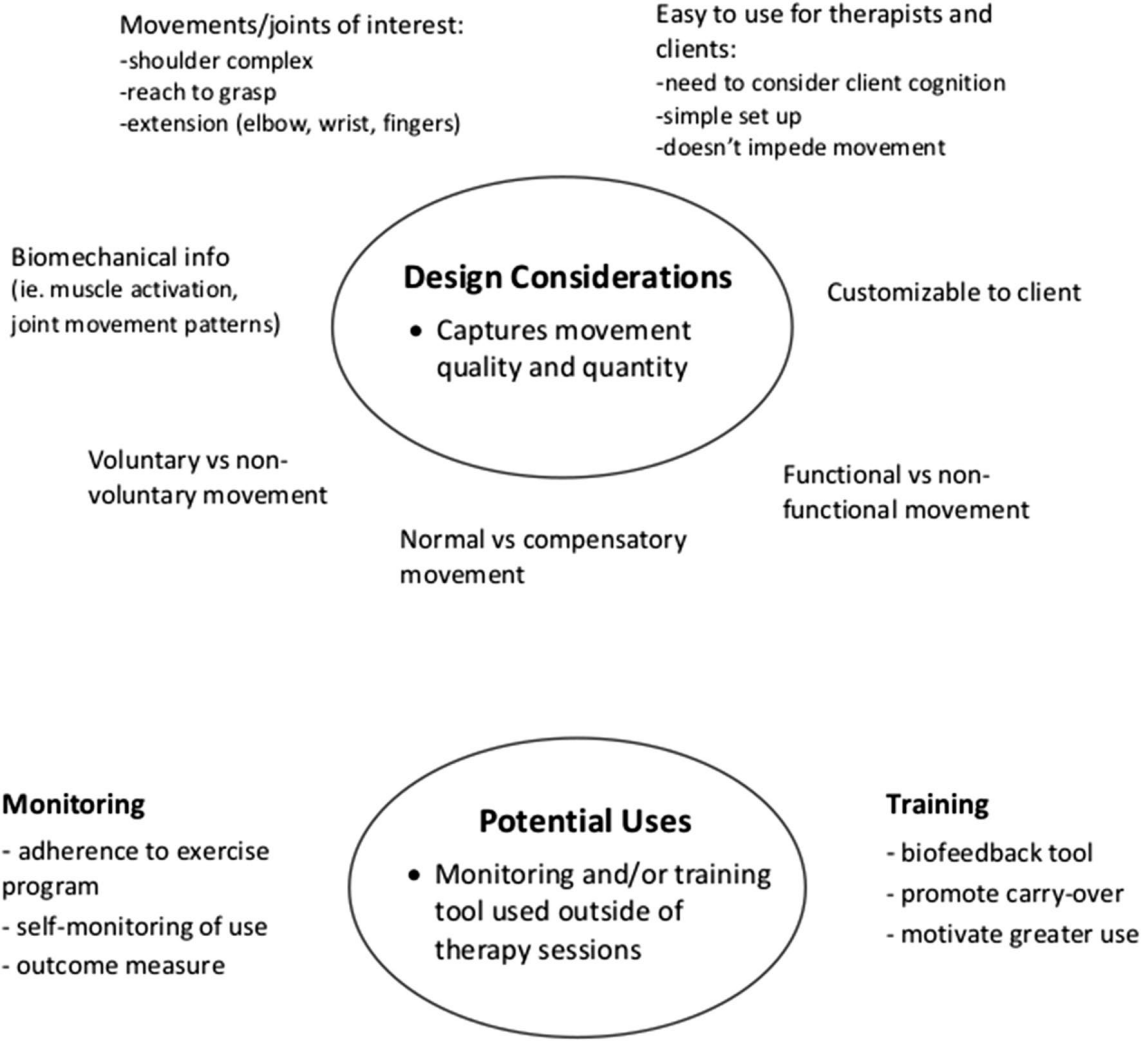
Summary of suggested design considerations and potential uses for a prospective wearable device

This study explored the perceptions of wearable devices for tracking upper limb activity post stroke from the perspective of occupational and physical therapists. Moreover, this is the first study that has investigated clinicians’ perceptions of wearable devices that specifically capture upper limb activity. Focus group discussions revealed many important considerations for the design and potential incorporation of wearable devices for tracking upper limb activity post stroke into clinical practice (Fig. 1).

### Quality and quantity of use outside of therapy

Therapists across the inpatient to chronic stroke rehabilitation continuum agreed that a device that could capture both how much and how well the client is using their affected limb outside of therapy is ideal. However, many therapists also felt a wearable device that only captured how much the arm was being used would still benefit clients who had functional use of their upper limb. For instance, monitoring adherence to an exercise program and motivating greater affected upper limb use through goal setting and promotion of self-monitoring were two potential uses for a wearable device identified in the focus groups. Importantly, these identified potential uses aligned with the common goal of maximizing functional use of the affected upper limb for individuals with some motor return identified by therapists. Moreover, the identified potential uses of self-monitoring and motivating greater use of the affected arm also align with a growing interest among rehabilitation researchers in the use of wearable devices to promote greater upper limb use [17] and some preliminary evidence to suggest that feedback from wearable devices may increase upper limb movement practice [18, 19]. If the evidence accumulates regarding wearable devices potential for increasing functional use of the upper limb then findings from these focus groups suggest that therapists’ interest in wearable devices that provide quantitative information on arm use and therapists’ stated priority of maximizing functional use may help facilitate future knowledge translation efforts.

The salience of movement quality across all focus groups was not an unexpected finding. The general goal of upper limb rehabilitation according to the Canadian Stroke Guidelines is to “enhance motor control and restore sensorimotor function” [20]. Therapists stated that devices able to capture movement quality could potentially promote carry over of the movement quality emphasized during therapy sessions. Thus, wearable devices able to capture movement quality might be more appealing to therapists as they are seen as an extension to their current practice. Indeed, a previous focus group examining perceptions of wearable robotic devices for the upper limb following stroke found that therapists had a desire for devices that complement traditional therapy [13]. In addition, a survey-based study of occupational and physical therapists in a large Canadian rehabilitation centre found that therapists’ performance expectations, defined as the degree to which they believe that the technology will help them achieve work and client goals, was the factor most related to their intention to use a new technology [12]. Finally, devices able to reliably capture upper limb movement quality may help overcome a concern about strengthening compensatory movement patterns, which was a barrier to greater exercise or activity prescriptions identified by many therapists in the inpatient setting.

Therapists identified many movements along the reach to grasp continuum such as scapular control and extension at the elbow, wrist and fingers on which they focus. These identified movements are consistent with clinical prediction tools that suggest that early shoulder abduction and finger extension are predictive of greater upper limb functional recovery [21]. In addition, therapists’ desire to distinguish between normal vs compensatory movement patters aligns with recommendations from the Stroke Recovery and Rehabilitation Roundtable for greater use of kinematic measurements in stroke recovery trials to distinguish between motor restitution vs compensation [22]. Although numerous research studies have reported the successful ability of various wearable systems to capture upper limb movement quality after stroke, there is still need for more research to make these systems appropriate for clinical use [9]. Findings from our study demonstrate clinician support for these research endeavors.

### User-friendly

Therapists highlighted the heterogeneity observed in clients’ clinical presentation and other client-specific personal and social factors. Client variability was also identified as a reason for their difficulty in identifying a core set of movements that a device should capture. Variability among stroke clients were emergent themes from recent focus group studies that examined clinicians’ perceptions of upper limb robotics and lower limb wearable technologies [13, 23]. The issue of heterogeneity in the field of stroke rehabilitation research has been documented extensively with greater calls for the field to determine the most appropriate target population and timing of treatments under study [24]. The questions of ‘who for’ and ‘when’ applies equally to the development and research of wearable systems for capturing upper limb activity post stroke. This study ascertained clinicians’ perceptions of wearable devices for people with some reach and grasp ability. Thus, clinicians might have different opinions when considering wearable devices for individuals with more severe impairments. In addition, therapists in our focus groups working in the inpatient setting expressed less interest in using a wearable device to monitor adherence to homework than outpatient therapists. Inpatient therapists in our study also reported greater challenge and concerns related to homework prescription. This finding suggests that using a device to promote greater amounts of movement practice may be less of a priority for inpatient therapists compared to outpatient therapists. However, this finding should be treated with caution as participants were not directly asked about their beliefs about movement intensity.

Finally, therapists highlighted the importance of ease of use for the clinical adoption of any device. Interestingly, perceived ease of use was not found to be an important factor in the explanation of technology use intentions in the study by Liu et al. [12]; however, our finding is consistent with two other studies which found that ease of set up was important for clinical adoption [11, 13].

### Limitations and future research directions

One major limitation of this study is that individuals with stroke or their caregivers were not included. Future studies examining important features from the perspective of the end-user of wearable devices that track upper limb activity are thus warranted. In addition, focus groups were often conducted during participants’ lunch hour. Time pressures often limited the moderator from probing deeper into treatment philosophies underpinning their views about future wearable devices. Finally, focus groups were conducted in 2015 and thus clinicians’ treatment practices and beliefs may have changed since then. It should be noted however that research suggests the implementation of research knowledge into clinical practice often takes much longer than six years [25].

## Conclusion

Therapists reported an interest in using wearable devices to capture upper limb activity outside of therapy sessions for individuals with some reach and grasp ability. Devices that are easy to use and capture quality and quantity may result in greater uptake in the clinical setting. Future studies examining acceptability of wearable devices for tracking upper limb activity from the perspective of individuals with stroke are needed.

## Supplementary Information

**Additional file 1. **Focus group guide.

**Additional file 2.** Final coding guide.

## Data Availability

Focus groups transcripts and audio files are not publicly available due to the risk of identifying the study participants.
