# Supplementary material for: Clinicians’ perceptions of a potential wearable device for capturing upper limb activity post-stroke: a qualitative focus group study
Source: J Neuroeng Rehabil. 2021 Sep 8;18:135. doi: 10.1186/s12984-021-00927-y (PMC8425094; doi:10.1186/s12984-021-00927-y)
Supplement: Supplementary file 1 — Additional file 1. Focus group guide. [file 12984_2021_927_MOESM1_ESM.docx]

**Focus Group Guide**

Intros, explanation of process and preliminary info

- Thank participants for coming and explain purpose:
  - Today we would like to talk to you about your experiences treating people with upper limb impairments after stroke and we are hoping to understand if and how a wearable device that captures upper limb activity might be useful in your practice.
- Introduce self and observer
- Explain focus group process
  - I just want to remind you that the focus group will be tape recorded so I can go back and more thoroughly process what was discussed today. The information you provide is confidential and thus we will not be using your real names or identifying information without your permission. Observer is here to assist in moderating and taking some notes.

Part 1: Description of current upper limb practice

I am just going to start the session by trying to get an overall picture about what upper limb rehabilitation looks like for many of your clients.

1. Can you describe some examples of the upper limb goals you create with your clients?
   1. Prompt: Sometimes it is the case that people will regain some function but they don’t necessarily use their affected limb to their ability (ie. the learned non-use concept). I am wondering if you ever create upper limb goals that involve how your clients can use the limb? Can you provide some examples?
2. Can you provide some examples of the type of homework tasks you might assign your clients?
   1. Prompt: What type of exercises or activities do you ask participants to do in between sessions?
3. It is sometimes the case that people with stroke will regain some function but they don’t necessarily use their affected limb. Can you provide some examples of how you encourage your patients to use their upper limbs more?

Part 2 Questions: Understanding clinician opinions about a sensor (without knowledge of prototype)

We are working with engineers to design a wearable device worn at the wrist that can track arm and hand movements either outside or inside a therapy session. Essentially we want it to count certain arm and/or hand movements to provide us with an objective measure of activity. We are envisioning this device would be suitable for clients with mild to moderate impairment (ie. they are at least able to reach). We are hoping to get your opinions about if and how this could be useful for your practice and what features you would like a wearable device to have.

1. What do you think about a wearable device that could track arm and hand movements?
   1. Prompt: What is your opinion about its usefulness in your clinical practice?
   2. Prompt: How might you use it during a therapy session?
   3. Prompt: How might you use it outside of a therapy session? Ie. giving it to clients to wear outside of therapy time or to check adherence to exercise program?
   4. Prompt: Would some sort of device that provides feedback of client’s activity be useful?
   5. Prompt: Would it be useful for helping to progress homework tasks (ie. keeping track of the repetitions of certain movements)
   6. Prompt: What do you think about using a device inside or outside of therapy to encourage clients to attain a certain number of arm movements?
   7. Prompt: Would anyone be uncomfortable with using a device to measure use, either during or outside of therapy time? ie. too prescriptive
2. If you were designing this sensor, what would you like the device to be able to do?
   1. Prompt: What arm and hand movements do you think it should count?
   2. Prompt: What types of things would you want it not to count (ie. particular compensations)?
   3. Prompt: Would you be interested in capturing use over a day or to capture particular activities? Or both?
   4. Prompt: Would you be interested in knowing how well someone approximates a particular activity? How might you use this information?
3. If you were designing this sensor, what features would you like it to have?
   1. Prompt: Would you like there to be separate modes for client vs therapist (ie. therapist mode captures more varied movements?)
   2. Prompt: How would the two modes be different?
   3. Prompt: What information would you like to see in the display?

Part 3: Understanding clinicians’ opinions about sensor (with knowledge of prototype)

The current prototype of the sensor is a strap with force myography sensors along it (can show them one). The force myography sensors measure the change in diameter of a muscle or tendon when they contract. The engineers have developed an algorithm using this technology to tell us when the hand is grasping something and when it isn’t. It then essentially counts the number of “grasps” a client performs.

1. What do you think about this sensor that counts number of grasps?
   1. Prompt: Do you think this information would be useful?
   2. Prompt: Would you need it to collect more information (ie. shoulder movements)
2. Right now the sensor has to be calibrated to a specific person. Explain calibration. How do you feel about the calibration of the sensor?
   1. Prompt: Would this detract you from using it?
   2. Prompt: What amount of calibration would you be ok with? Provide examples if necessary (ie. once per person at the beginning of use than that’s all)
   3. Prompt: What frequency and amount of time would be reasonable?
