# Supplementary material for: Clinicians’ perceptions of a potential wearable device for capturing upper limb activity post-stroke: a qualitative focus group study
Source: J Neuroeng Rehabil. 2021 Sep 8;18:135. doi: 10.1186/s12984-021-00927-y (PMC8425094; doi:10.1186/s12984-021-00927-y)
Supplement: Supplementary file 2 — Additional file 2. Final coding guide. [file 12984_2021_927_MOESM2_ESM.docx]

**Wearable Sensors to capture UL activity Focus Groups: Code Guide Final**

| **#** | **Code** | **Description** |
| --- | --- | --- |
| **1** | Considering level of impairment-practice | Therapists reporting clients’ level of motor, cognitive or other impairment (ie. fatigue) severity to qualify their clinical decisions |
| **2** | Considering level of impairment-device | Therapists reporting clients’ level of motor, cognitive or other impairment (ie. fatigue) severity to qualify their opinions about using a device |
| **3** | Considering client factors | Therapists discussing how client factors such as hand dominance, time post stroke and client preferences impact their decision making or how it relates to their perceptions of a potential wearable device |
| **4** | Promoting functional use of UL | Therapists discussing ways in which they promote, maximize or attempt to increase clients’ functional use of their affected upper limb |
| **5** | Highlighting complexity of UL rehab | Therapists stating ways in which UL rehabilitation and/or designing a wearable device for the UL is complex or difficult |
| **6** | Prescribing homework is tricky | Therapists describing the specific ways they prescribe homework (or don’t) that accounts for the varied needs of their clients and suggests the challenging nature of the task |
| **7** | Wanting a customizable device | Therapists describing how they would like a device to be customizable |
| **8** | Managing impairments | Therapists discussing how managing impairments such as tone, pain, strength, and fatigue are used in their practice |
| **9** | Using sensory stimulation | Therapists describing when they use sensory stimulation in their practice |
| **10** | Using supported practice | Therapists discussing ways in which they use caregivers or rehabilitation staff to support upper limb rehabilitation or how they envision a caregiver could assist with wearable device management |
| **11** | Capturing movement quantity can be useful | Therapists stating that capturing movement quantity would be useful to their practice (at least in part) |
| **12** | Stressing importance of capturing functional use | Therapists asserting that a device would ideally capture “functional” use of that limb (ie. movement of a limb that is helping to accomplish a task) |
| **13** | Debating usefulness of capturing quantity | Therapists discussing whether capturing quantity alone or at all would be useful |
| **14** | Using tool for biofeedback | Therapists discussing using a potential wearable device to educate clients on how to move their affected UL |
| **15** | Promoting carry over | Therapists describing desire for clients to carry over what they have learned during therapy sessions into their movement outside of therapy |
| **16** | Desiring knowledge of movement/activity outside of sessions | Therapists expressing a desire for knowledge of how and how much clients are moving outside of therapy |
| **17** | Motivating greater use | Therapists describing how a wearable device could be used to motivate greater affected UL use |
| **18** | Stressing importance of movement quality | Therapists describing an interest in promoting greater UL movement quality which includes approximating more normal movement patterns. |
| **19** | Wanting info about biomechanics | Therapists describing a desire for a device to provide biomechanical information about the UL which includes joint angles, muscle activation and muscle forces |
| **20** | Worrying about movement quality outside of therapy | Therapists expressing concern over how clients are moving their UL outside of therapy |
| **21** | Identifying compensatory movement | Therapists describing common undesirable UL movement patterns observed in their clients with stroke |
| **22** | Monitoring adherence | Therapists describing how they monitor clients’ adherence to homework or how a device could be used to monitor adherence to homework |
| **23** | Monitoring adherence-disagree | Therapists stating they do not believe a wearable device should be used to monitor adherence to homework |
| **24** | Desiring voluntary movement | Therapists stating how a device could ideally differentiate between voluntary and passive movement |
| **25** | Identifying specific movements of interest: extension | Therapists describing how this movement of interest is incorporated into their current therapy or identifying this as a movement they would like a future device to capture |
| **26** | Identifying specific movements of interest: shoulder | Therapists describing how this movement of interest is incorporated into their current therapy or identifying this as a movement they would like a future device to capture |
| **27** | Identifying specific movements of interest: reach to grasp | Therapists describing how this movement of interest is incorporated into their current therapy or identifying this as a movement they would like a future device to capture |
| **28** | Identifying specific movements of interest: grasp and release | Therapists describing how this movement of interest is incorporated into their current therapy or identifying this as a movement they would like a future device to capture |
| **29** | Identifying movement of interest: fixed posture | Therapists describing how this movement of interest is incorporated into their current therapy or identifying this as a movement they would like a future device to capture |
| **30** | Debating specific movements of interest | Therapists debating whether a specific movement is important to capture or not |
| **31** | Requiring ease of use | Therapists describing how they would want any future wearable device to be easy to use |
| **32** | Providing concrete info | Therapists describing how a potential wearable device could provide concrete information to the therapist and/or client about client’s UL use |
| **33** | Increasing awareness of limb | Therapists describing how they facilitate an increase in UL awareness in their practice |
| 34 | Context code: caseload | Describing make up of caseload |
| 35 | Clarifying question | Asking moderator or other participant a clarification question |
| 36 | Unrelated discussion | Discussion unrelated to research questions |
